# Multiple Factors Involved in Bone Damage Caused by Chikungunya Virus Infection

**DOI:** 10.3390/ijms241713087

**Published:** 2023-08-23

**Authors:** Amanda M. Avila-Trejo, Lorena I. Rodríguez-Páez, Verónica Alcántara-Farfán, J. Leopoldo Aguilar-Faisal

**Affiliations:** 1 Laboratorio de Bioquímica Farmacológica, Departamento de Bioquímica, Escuela Nacional de Ciencias Biológicas, Instituto Politécnico Nacional, Mexico City 11340, Mexico; ammarat.gdds@gmail.com (A.M.A.-T.); lorena_rpaez@yahoo.com.mx (L.I.R.-P.); veroalf@yahoo.com (V.A.-F.); 2 Laboratorio de Medicina de Conservación, Secretaría de Estudios de Posgrado e Investigación, Escuela Superior de Medicina, Instituto Politécnico Nacional, Mexico City 11340, Mexico

**Keywords:** chikungunya, alphavirus, bone damage, bone resorption

## Abstract

Chronic cases of chikungunya fever represent a public health problem in countries where the virus circulates. The disease is prolonged, in some cases, for years, resulting in disabling pain and bone erosion among other bone and joint problems. As time progresses, tissue damage is persistent, although the virus has not been found in blood or joints. The pathogenesis of these conditions has not been fully explained. Additionally, it has been considered that there are multiple factors that might intervene in the viral pathogenesis of the different conditions that develop. Other mechanisms involved in osteoarthritic diseases of non-viral origin could help explain how damage is produced in chronic conditions. The aim of this review is to analyze the molecular and cellular factors that could be involved in the tissue damage generated by different infectious conditions of the chikungunya virus.

## 1. Introduction

Chikungunya virus (CHIKV) is an arbovirus that was first isolated in 1952 in Tanzania, Africa, where it is endemic [1]. However, in recent years, epidemic outbreaks have allowed it to spread worldwide. Its main vectors are *Aedes aegypti* and *Aedes albopictus* [2]. The chikungunya virus has a single-stranded RNA with positive polarity that codes for two polyproteins. One contains the non-structural proteins (nsP1, nsP2, nsP3, and nsP4). The second one contains the structural proteins, including the capsid protein, E1, and E2, which form heterodimers and assemble the virus envelope [3].

Chikungunya virus belongs to the family *Togaviridae*, genus *Alphavirus*, which contains numerous animal and human pathogens. Furthermore, it can be divided into two major groups. These two major groups are based on the hypotheses of their origin and/or evolution and based on the main symptoms they produce. “New World” alphaviruses are those that produce encephalitis, such as Venezuelan Encephalitis Virus and Eastern Equine Encephalitis Virus; meanwhile, “Old World” ones produce infections with arthritogenic symptoms, such as Sindbis virus, Ross River virus, o’nyong-nyong virus, Semliki Forest virus, and chikungunya virus, which in humans, produces an infection known as chikungunya fever [4].

Chikungunya fever disease is characterized by symptoms, such as an acute fever above 39 °C, headache, vomiting, skin rashes, asthenia, severe muscle pain, and generalized and debilitating arthralgias that significantly affect the quality of people’s life. Symptoms may last for days or weeks in acute and subacute conditions; however, when they exceed three months, they are considered chronic [5]. It is estimated that more than 40% [6,7] of infected individuals develop a chronic state of the disease with arthritic characteristics, such as bone erosion, enthesopathies, periostitis, and persistent and debilitating joint pain [8,9], which is reflected in the population’s public health through disability-adjusted life years (DALYs) and the economic burden involved [10,11]. However, although bone damage in CHIKV infections has been documented, the mechanism involving progression and subsequent injury has not been fully defined.

**Figure 1:**
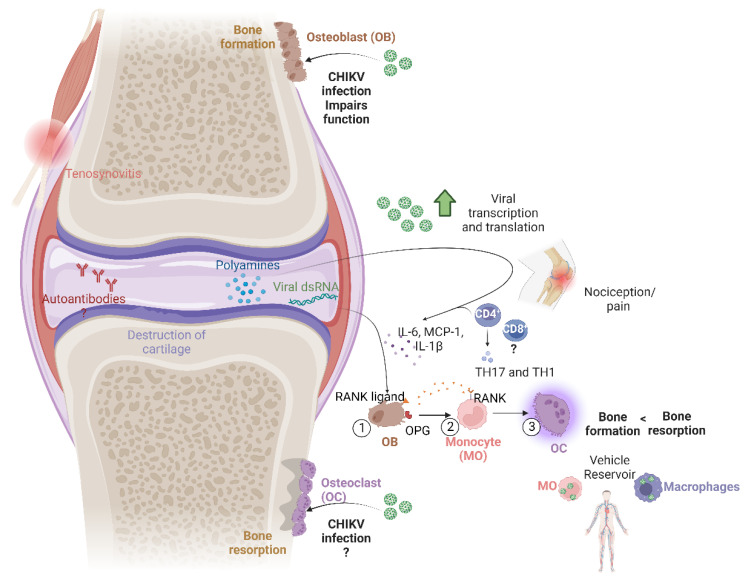
Multiple mechanisms associated with bone damage caused by chikungunya virus infection (created with BioRender.com).

This review will focus on different cellular and molecular factors that have been reported to be involved in the processes of bone erosion in a chikungunya virus infection. It will also analyze other possible mechanisms that could occur when people develop a chronic stage of the disease, as shown in Figure 1. Finally, it will review the latest treatment proposals for this manifestation, given that this disease does not yet have a specific antiviral.

## 2. Bone Damage and Its Mechanisms

Bone erosion consists of cortical bone disruptions, and sometimes, it can include a decrease in trabecular bone [12]. Normally, the bone system maintains a balance between bone production and bone resorption via osteoblasts and osteoclasts, respectively. But an alteration to this balance favors osteoclastogenic activity and thus bone loss [13].

In the case of chikungunya fever and other arthritogenic alphavirus diseases, inflammatory processes are characterized by IL-6, MCP-1, and IL-1 production, implying an osteoclast differentiation increase and osteoblast functionality damage. Osteoclasts are cells of the monocytic lineage, which are attached to the periosteum surface and generate an acidified environment extending from the undulating basal portion into the bone. This zone acidification, which is mediated by proton pumps, allows for bone demineralization, and in conjunction with the secretion of matrix metalloproteinases and cathepsin K, degrades the bone matrix [14].

## 3. Cell Signaling in Bone Resorption

The relationship between osteoblasts and osteoclasts is marked by a regulatory interaction. Osteoblasts express the NF-κB ligand receptor activator (RANKL), while its receptor is expressed on the monocytic osteoclast precursor (RANK). When RANKL and RANK bind together, osteoclast differentiation and activation are stimulated [15].

To regulate this process, osteoblasts also produce osteoprotegerin (OPG), a regulatory factor, which binds to RANKL to prevent it from binding to its receptor, limiting osteoclastogenesis [16]. Osteoprotegerin is the only protein member of the TNF receptor family that can be present both as a soluble form and on the membrane surface of osteoblasts. Due to its high affinity for heparan sulfate in this cellular area, it binds to it and is immobilized. After being fixed to the membrane, OPG undergoes a fundamental conformational change in its function against bone resorption, becoming a more compact and less flexible molecule [17,18].

There are still questions remaining about the roles of RANKL and OPG in bone degradation. RANKL has a soluble form, and although it does not have a direct impact on bone loss, its absence decreases the number of osteoclasts and increases bone mass [19]. In the case of OPG, questions arise as it is unusually present in the osteoclast membrane where the soluble form of RANKL is, and it can bind to it [20]. It cannot be ruled out that the virus possesses pathogenesis mechanisms directly linked to the interaction with these proteins. It has also been demonstrated that viral proteins are able to bind to other proteins, thus regulating their function/expression.

During the acute phase of CHIKV infection, the level of viremia can determine specific patterns of pro-inflammatory cytokines associated with disease severity. In the chronic phase, it has been found that levels of IL-6 and granulocyte macrophage colony-stimulating factor (GMCS-F) may explain persistent arthralgia and bone damage. These cytokines are involved in differentiating osteoclasts from monocytic precursors, but the role of IL-6 is still controversial. Recent research has shed new light on the role of IL-6 in osteoclast activity and bone resorption. While it was previously believed that IL-6 primarily stimulates osteoblastic/stromal cells to produce RANKL, which then induced the differentiation of osteoclast precursors into mature osteoclasts, evidence suggests that IL-6 has direct effects on osteoclast formation. A RANKL-independent mechanism for IL-6-mediated osteoclast formation has been reported. Additionally, IL-6 has been shown to inhibit RANKL-induced osteoclast formation, further complicating our understanding of its role in osteoclastogenesis. To better understand the impact of IL-6 on osteoclast formation, researchers have examined the effects of IL-6 deficiency in mice. They found that the depletion of IL-6 resulted in increased numbers of osteoclasts with reduced resorptive activity, indicating that IL-6 may have separate effects on the number and function of osteoclasts [21]. Also, recent studies have suggested that IL-6 and the IL-6 receptor enhance the expression of RANKL mRNA and the RANKL/OPG expression ratio [22]. However, it should be noted that other studies have demonstrated that IL-6 promotes low levels of RANKL-induced osteoclastic differentiation in mouse BMMs through a trans-signaling pathway [23], and the expression levels of IL1β, TNF, IL12p40, and MMP3 are significantly lower in this osteoclast type [24]. Despite these new findings, the precise target cells affected by IL-6 and its role in osteoclastogenesis remain debatable.

## 4. Persistence of Viral RNA and Proteins in Chronic Cases

One of the main problems in understanding the virus pathogenesis related to bone damage is determining whether a persistent infection is responsible for bone erosion or not. In this regard, there is still no definitive answer. In chronic patients, the presence of viral RNA and infectious viral particles in synovial fluid and blood has not been demonstrated [8,11,25]. However, in an individual who developed a chronic 18-month course phase, evidence was found of a persistent active infection in the synovial perivascular macrophages [26].

Chikungunya virus is a single-stranded ribonucleic acid (ssRNA); however, it synthesizes double-stranded ribonucleic acid (dsRNA) for the formation of replication spherules in the inner plasma membrane during the early stages of alphavirus infections, even before the production of virus structural proteins [27,28]. It is important to emphasize that the prolonged presence of viral RNA in the affected tissue not only contributes to the inflammatory process mediated by the production of TNF-α, IFNs, and IL-6, but it has also been shown that viral origin dsRNA in serum and synovial fluid correlates with bone erosion due to a mechanism involving NF-κB activation in monocytes/macrophages [29,30,31]. In chronic phases, viral RNA has not been found in synovial fluid via RT-PCR and RNA in situ hybridization. However, the use of histological tissue sections in combination with immunohistochemistry has been able to detect viral RNA in spleen macrophages in non-human primates [32] and in skin and muscle fibroblasts [33]. Therefore, it is necessary to continue with viral dsRNA research as the main cause of persistent inflammation due to PAMP activation in joints where periosteal erosion is present in chronic conditions. Different techniques are currently being analyzed for detecting the presence of viral dsRNA, such as the use of monoclonal antibodies and confocal microscopy, Northwestern blot analysis, and even in vivo visualization using transgenics [34,35].

In studies in mice, E1 RNA and the capsid protein were found late through RT-PCR and immunohistochemistry, respectively, in an infected mouse paw; this could suggest the active translation of viral proteins given the viral RNA presence in the chronic phase (60 days post-infection in mice). However, this hypothesis contrasts the fact that it was not possible to isolate infectious viral particles after day 14, where isolation was attempted from macerated tissue, after being digested with collagenase, or through supernatant collection, etc. [36]. This aligns with another study using a mouse model, where RT-qPCR detected the virus in immunocompetent mice for up to 12 weeks without proving they were infectious particles [37]. Therefore, it can be inferred that the tissues where viral RNA has been found are related to the pathogenesis, which involves immune processes with the participation of recruited macrophages, neutrophils, and even eosinophils. Phagocytosis and the formation of neutrophil extracellular traps [38] could contribute to the absence of viral RNA in the synovial fluid. However, the presence of viral RNA structural proteins without infectious particles in between could support the hypothesis that the former participate in storing, but do not actively replicate.

## 5. The Role of the Immune Response

There are different types of cells in the body that participate in viral pathogenesis; however, in bone erosion, immune system cell participation stands out due to the production of cytokines and chemokines that generate a proinflammatory environment.

The innate immune response induced by CHIKV occurs in the early stages of infection since the main goal is to prevent viral replication. Additionally, in regard to the pro-inflammatory environment formation, different types of interferons (IFN-/) are produced, leading to the consequent expression of IFN-stimulated genes (ISGs). Among these IFN-stimulated genes, we can highlight SAT1, a gene that contributes to the restriction of viral replication through the depletion of polyamine pools. Polyamines are derived from ornithine and synthesized through a complex enzymatic pathway. These molecules are essential for many cellular processes and play a crucial role in the life cycle of the chikungunya virus. Studies have shown that when spermidine and spermine levels are reduced through the induction of spermidine/spermine N1-acetyltransferase (SAT1), CHIKV replication is restricted. This restriction occurs both in vitro and in vivo due to impaired viral translation and RNA replication. This suggests that SAT1 and polyamine depletion could be promising avenues for antiviral therapies [39].

High levels of polyamines have been found in the synovial fluid of arthritic patients; this might be caused by the activity of the enzyme ornithine decarboxylase, which is also increased at the sites of inflammation. The enzyme ornithine decarboxylase is responsible for the synthesis of the following polyamines: putrescine, spermidine, and spermine, which are involved in the development of nociception in the joints [40]. In rheumatoid arthritis, synovial fibroblasts show aggressive behavior and express high levels of SAT1, which decreases the concentration of polyamines. As an opposite development, DNA demethylation occurs through the addition of S-adenosyl methionine as a precursor for polyamine synthesis; this methylation leads to the expression of endogenous retroviral sequences, which are associated with inflammation and joint destruction in different diseases [41,42,43,44,45].

In chikungunya virus infections, the presence of retroviral sequences has not been demonstrated. However, the inflammatory process is present at different stages of the disease. This inflammation generates an increase in the concentration of polyamines, benefiting the processes of viral transcription and translation. On the other hand, the antiviral role of IFN is inhibited by its interaction with the viral non-structural protein nsP2; this interaction causes the ISGs to not be stimulated and the expression of SAT1 to decrease. It is important to highlight that even though the virus presents antiviral strategies with respect to the IFN, it fails to completely block its activity. It has been reported that the virus mortality rates are low [46,47]. In IFN-I signaling experiments with genetically modified mice, the absence of IFN-I meant rapid death from infection [48]. Ultimately, the virus’s need for polyamines and the different regulatory pathways involved in their upregulation and downregulation make them a potential therapeutic target.

Regarding the adaptive immune response, antibodies produced by plasma cells can play different roles in chronic disease. In addition to the hypothesis of the presence of autoantibodies in the autoimmune process that will be discussed later, the presence of long-lasting IgM antibodies has been documented [8,10]. This long-lasting presence of IgM antibodies could suggest the persistence of viral antigens in different tissues, although a statistical correlation with chronic disease has not yet been determined [49]. On the other hand, the late production of the IgG3 subclass is associated with low viremia, but the predominantly evolves into chronic disease [50].

Meanwhile, T lymphocytes actively participate in the inflammatory infiltrates present in virus-infected joints. It has been demonstrated that the CD4+ lymphocytes are actively involved in joint pain and inflammation through the production of cytokines and chemokines related to Th17 (IL-1, IL-6, and IL-17) and Th1 (IFN-, TNF- α, IL-12, IL-15, IL-18, IP-10, Mig, MIP-1α, and MIP-1) profiles [51]. In the case of memory CD8+ T lymphocytes, their participation in the processes of bone erosion in rheumatoid arthritis has been reported [52]. Although the mechanism by which they act has not been elucidated, it is likely that they might be involved in the exacerbated bone resorption caused by a CHIKV infection. It should be noted that the T-lymphocyte response, which is detected in chronic cases [53], is age dependent and has a more pronounced response in older individuals [32].

Monocytes and macrophages are the predominant cells in the inflammatory infiltrate of chikungunya fever. Macrophages act as virus reservoirs in subacute phases, although this has not been demonstrated in chronic human cases [54]. Cytokine production via macrophages includes prostaglandins, IL-6, and TNF-α and favors tissue damage caused by inflammation in the joint [55]. An important fact in viral pathogenesis is that the monocytes are also capable of replicating the virus, acting as a vehicle [56]. It is important to mention that monocytes might have a role as osteoclastic precursors; this role would directly indicate their involvement in bone resorption. The damage that a CHIKV infection produces depends on the cytokines and chemokines generated in the process. However, as part of the mechanism by which bone resorption occurs, the monocyte chemoattractant protein (MCP-1) is elevated, thus recruiting monocytes for differentiation into osteoclasts and promoting exacerbated bone loss [57].

Osteoblasts can be infected with the virus, stimulating IL-6 production and altering the OPG/RANKL ratio. This would favor the differentiation of monocytic precursors into osteoclasts, which are the cells that resorb bone in erosive processes [58,59].

## 6. Comorbidities

It is important to consider the hypothesis that the presence of other diseases affecting bones, joints, or a part of the metabolism prior to infection with chikungunya might increase arthritis damage or favor the development of a chronic disease [54,60,61]. In contrast, it has been reported that patients who developed a chronic stage of the disease and present with damage through radiographic images had no history of joint disease [62]. The lack of a consensus on the relevance of prior diseases in the progression to chronic disease highlights the need for further studies with individuals who continue to have symptoms three months post-infection to understand and establish the relationship that these symptoms might have with other comorbidities.

## 7. Genetic Factors

Individuals might present with a specific genetic factor that favors the development of a chronic condition and leads to bone damage. However, questions are raised related to the infection itself. It has been observed that up to 95% of people develop chikungunya fever after being infected through the bite of a mosquito vector [62]. It has also been reported that high rates of asymptomatic infections can occur, exceeding 40% of all infected persons [63]. Therefore, if there is a certain genetic susceptibility to develop symptoms, it is also feasible that there are factors that determine the risk of presenting with bone erosions in chronic cases. A study case of a group of infected families in India found that some susceptibility between blood groups exists, while Rh-negative individuals were resistant to infection [64]. In contrast, it has been reported that in the Indian population, HLA-DRB1*11 and HLA-DRB1*11-HLA-DQB1*03 haplotypes are associated with infection resistance, while HLA-DRB1*04-HLA-DQB1*03 has been linked to susceptibility to developing the disease [65].

Regarding the severity of symptoms, which might include arthritis and bone damage, a non-coding SNP, rs6552950, present in the TLR3 gene has been reported. Additionally, with regard to chronicity, a population from the Reunion Islands was studied, where it was demonstrated that more than 60% of patients who had arthritis caused by the virus also had HLA-DRB1*01 or HLA- DRB1*04 alleles, pointing out the important role of HLA class II in virus infection [66]. There are other studies that specifically looked at rheumatoid arthritis, alleles, and SNPs, such as those that influence RANK/OPG/RANKL and are directly linked to erosion, but they did not consider infection with the chikungunya virus [67].

Similar to the fact that genetic variations have been found to be associated or not with the development of symptoms, they can also be associated with autoimmunity processes. This implies two hypotheses: the first one being that the pressure on the human genome given by pathogens has produced a defense gene selection, which increases the risk of developing autoimmune diseases, and the latter being one of the proposed causes of chronic conditions in CHIKV infections. The second hypothesis implies that viral infection activates autoimmunity mechanisms through molecular mimicry, epitope spreading, bystanders, or cryptic antigens. These mechanisms have already been reported in other cases of viral infections as elements that induce autoimmune processes and even mimic the symptoms of other diseases, such as rheumatoid arthritis or osteoarthritis [68,69,70].

## 8. Viral Arthritis Markers

The most common presentation of viral arthritis is acute-onset polyarticular arthritis. Although viruses cause only a small proportion of cases of acute arthritis, the differentiation of virally mediated arthritis from primary rheumatological disease is important as it influences the subsequent management. It is interesting to note that viral arthritis is a condition that is reported all over the world, but the actual incidence and prevalence of it are not well known. This could be because many different viruses can cause arthritis syndrome, as well as the fact that the illness is often self-limited. Rates of viral arthritis are much higher in adults than in children, with parvovirus B19 being the most common cause in children. In most cases of viral arthritis, the exact mechanisms causing it are not well understood. However, viruses can cause joint symptoms in various ways, including through direct invasion of the joint, immune complex formation, and immune modulation that causes chronic inflammation. It is thought that many viruses that cause arthritis take up residence in the joint synovium, which then leads to the recruitment of inflammatory cells and continuation of the inflammation cascade [71].

Most arthritogenic viral infections share the presence of specific biomarkers that have also been associated with rheumatoid arthritis (RA). A parvovirus B19V infection can affect the cytokine levels in the plasma of healthy individuals and RA (rheumatoid arthritis) patients differently. In RA patients, the production of IFN_γ_ is decreased and plasma IL-4 levels are raised, which can lead to lower antiviral clearance [72]. Hepatitis-B-virus-associated non-rheumatoid arthritis can also develop in affected joints in different patterns. Antinuclear antibodies (ANAs) and the rheumatoid factor (RF) are determined in approximately 10% and 25% of such patients, respectively, while the complement is reduced in almost half of them. In a Hepatitis C virus infection, cryoglobulins, the RF, and ANAs are detected in 43%, 60%, and 20% of cases, respectively [73]. Chronic HIV infection can increase the risk of systemic autoimmunity, with anticyclic citrullinated peptide antibodies detected in up to 15% of HIV-infected patients with advanced disease [74]. It has been suggested that RA may be caused by specific strains of EBV, with the citrullinated EBNA-2 peptide recognized specifically by RA sera [75]. Biomarkers of chikungunya-positive patients have revealed increased levels of the CRP (C-reactive protein), anticyclic citrullinated peptide (anti-CCP) antibody, soluble interleukin-2 receptor (sIL-2R), cartilage oligomeric matrix protein (COMP), hepatic ALT (alanine aminotransferase), AST (aspartate aminotransferase), ALP (alkaline phosphatase), albumin, and bilirubin among arthritic patients [76]. Finally, non-specific constitutional arthralgia is the most common joint manifestation during ZIKV infection, but it resolves in two weeks in >90% of cases, with no evidence of chronic rheumatic manifestations [77].

## 9. Autoimmunity and Bone Erosion

The different mechanisms reviewed above have included the presence of the virus (RNA and/or viral proteins [25,26,27,28,29,30,31,32,33,34,35]), the role of immune cells, the production of cytokines and chemokines, and the presence of certain SNPs. All of these suggest that the progression of the clinical picture of chikungunya fever to the chronic phase, which includes arthritis and bone damage, results from the post-infection process. The inflammatory response may have similarities with other rheumatic inflammatory autoimmune diseases [78].

However, these mechanisms are controversial since no autoantigens have been found and there are disagreements regarding other biological markers of these diseases. It has been reported that more than 50% of people who present this mimicry with other arthralgia diseases show systemic inflammation with the presence of a rheumatoid factor, in addition to the presence of some HLA alleles mentioned above and anticyclic citrullinated peptide antibodies [70,71,72,73,74,75,76,77,78,79]. On the other hand, two studies carried out in Colombia showed that more than 95% of the cases were found to be negative for anticyclic citrullinated peptide antibodies and the rheumatoid factor [80,81]. Therefore, a deeper and broader analysis of the subject is also required.

Among the other differences that stand out is the distinction of chemotaxis in arthralgia autoimmune diseases, where the synovial infiltrates contain neutrophils, while in chronic conditions caused by chikungunya fever, these are rather scarce, which allows for the production of monocytes and macrophages [82].

In the case of autoantibodies, the discrepancies continue. While some studies did not find any antinuclear antibodies, others had inconclusive results [38,41]. There is only one report where it was able to demonstrate the presence of antinuclear antibodies, but not anti-small nuclear ribonucleoproteins (snRNPs) [83].

## 10. Treatment Perspectives

**Figure 2:**
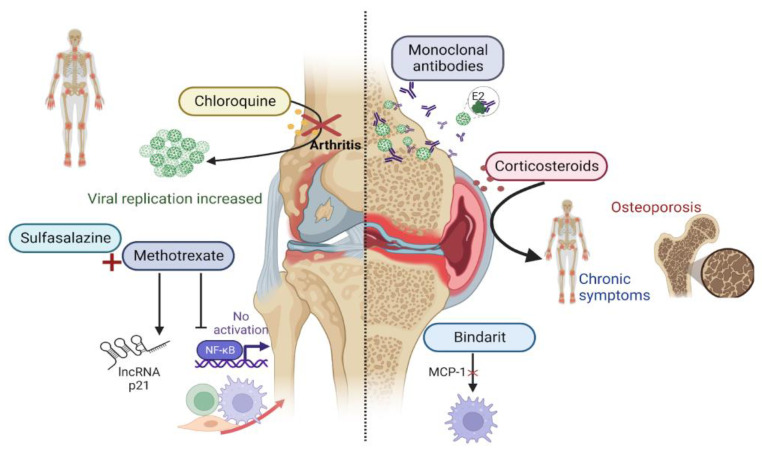
Treatments for chikungunya fever are non-specific and primarily focus on inhibiting inflammation pathways related to pain and decreasing viral replication (created with BioRender.com).

The treatment of acute cases produced by the virus is mainly oriented towards fever and joint pain control (Figure 2) [84]. In the case of bone erosion, which is produced by the chronic inflammation of destructive arthritis, drugs that are already used to treat other types of non-infectious arthritis have been studied [85,86,87,88]. This has been researched given the similarities in the pathogenic mechanisms between the two diseases [6]. Non-steroidal anti-inflammatory drugs are usually prescribed from the onset of infection. Although corticosteroids provide greater relief from pain and inflammation, it has been reported that their consumption might favor the return of arthritis and tenosynovitis [82]. In addition, complications can arise from the chronic consumption of corticosteroids, which include cataracts, glaucoma, diabetes mellitus, osteopenia, and osteoporosis [85,86].

As for other disease-modifying antirheumatic drugs, trials with chloroquine showed unsatisfactory results. These studies not only failed to decrease the duration of viremia and arthralgias, but also increased the rate of chronic arthralgias and viral replication [55,87]. In contrast, its derivative, hydroxychloroquine, has only shown an effect in combination with methotrexate. It has been reported that low doses of methotrexate reduce inflammation using its immunomodulatory activity without increasing viral replication [88]. In clinical studies, it has shown a decrease in the pain duration [88,89]. A combination of methotrexate and sulfasalazine, which is another drug that has also been evaluated in patients with chronic conditions, showed an improvement of 71.4% [89]. The variety of research carried out, including combined drugs and in some cases, no control groups, makes it difficult to obtain more accurate information about methotrexate performance in relation to disease chronicity.

Additionally, trials with monoclonal antibodies have been performed, but only for the acute phase of the disease [90]. Most of them have focused on E2 protein neutralization, which has been demonstrated as having a conserved region that favors viral persistence in joints and avoiding neutralization by antibodies. The neutralization of the E2 protein would decrease viral replication, avoiding the chronic phase of the process [91]. Immunotherapies using anti-IL-6, anti-TNF, and anti-RANKL, to try to avoid the damage produced by arthritic inflammation, have been proposed. Finally, the drug bindarit, which blocks MCP-1 signals, and pentosan polysulfate, which prevents cartilage thinning and the infiltration of immune system cells, can prevent bone erosion [92,93].

## 11. Conclusions

The most recent research on the chikungunya virus has allowed us to learn more about the factors involved in the processes of bone erosion and about the pathogenic relationship it has with other non-infectious arthritic diseases. However, there are contradictory reports about what is known regarding the virus. These contradictions are especially related to the persistence and the physiological and viral mechanisms that lead to chronic condition progression in certain individuals. There are also some contradictions in the findings of how arthritis development leads to bone erosion. All of the knowledge obtained on non-infectious arthritis and other alphaviral infections, as well as on chikungunya fever, has certainly contributed to our understanding of the disease. However, there are different diseases with particularities, and hence, there is a need for specific research regarding CHIKV infections. It is important to continue with these viral pathogenesis studies to elucidate and propose new therapeutic targets that can help to prevent exacerbated bone resorption from occurring and significantly impacting the life quality of people suffering from chronic chikungunya fever.
